# Epidemiological, humanistic and economic burden of dog-mediated rabies in India: a systematic review protocol

**DOI:** 10.12688/f1000research.28454.2

**Published:** 2021-05-12

**Authors:** Denny John, Abhishek Royal, Omesh Bharti

**Affiliations:** 1 Amrita Institute of Medical Sciences & Research Centre, Amrita Vishwa Vidyapeetham, Kochi, Kerala, 641112, India; 2 Faculty of Medicine, Public Health and Nursing, Universitas Gadjah Mada, Yogyakarta, Special Region of Yogyakarta, 55281, Indonesia; 3 Department of Health & Family Welfare, State Institute of Health and Family Welfare, Shimla, Himachal Pradesh, 171009, India

**Keywords:** Rabies, Dog bite, Systematic Review, Protocol, Burden of illness, Epidemiology, Humanistic burden, costs

## Abstract

**Background: **Rabies is a neglected zoonotic disease. It is transmitted through the bite of a rabid animal and dog bites are responsible for around 95% of human cases. The disease is almost always fatal after the onset of symptoms. It is an endemic and major public health problem in India with one-third of the global deaths reported from this country.

**Protocol: **This systematic review aims to estimate the epidemiological, humanistic and economic burden of dog-mediated rabies in India. Initially the existence of controlled descriptors in MeSH terms (such as 'Epidemiology', 'Rabies', 'Cost', 'Dog bite', 'Quality of Life', 'India' etc), and their synonyms (key words) was identified in MEDLINE, and were later combined with Boolean operators 'AND' and 'OR' to develop a detailed search strategy. Two independent reviewers will screen the titles and abstracts and select the studies as per the inclusion criteria. The selected studies will be assessed for their quality and risk of bias. Data will be extracted using standardized data extraction tools and will be synthesized for analysis. Disagreements that arise between the reviewers will be resolved through discussion, or with a third reviewer.

**Discussion: **This systematic review will be performed to critically examine relevant literature and report the epidemiological, humanistic and economic burden of dog-mediated rabies in Indian context. The findings will help in estimation of burden of the disease in India and expected to contribute in policy making and planning of the program and interventions in the country.

**Protocol registration:** PROSPERO ID: CRD4202021326

## Introduction

Rabies is a neglected zoonotic disease which results in 59,000 deaths per year across the globe ^1^. It is nearly 100% fatal after the onset of symptoms but could be prevented through timely access to post-exposure prophylaxis (PEP) following animal bites ^2, 3^. The administration of the recommended PEP following an exposure is guided through three World Health Organization exposure categories: I (no exposure), II (exposure) and III (severe exposure) ^4^. As dogs are responsible for more than 95% of human cases, the effective strategy also includes vaccination of dogs against rabies and pre-exposure prophylaxis (in the form of vaccination) for high risk individuals including veterinary healthcare workers, children and adults at risk ^3^.

Rabies is an endemic disease and major public health problem in India. It is prevalent across the entire country except in Andaman & Nicobar and Lakshadweep Islands. Annual human deaths due to rabies are estimated to be around 20,000 and the annual incidence of animal bites to be 1.7% (17.5 million per year) in India ^5^. The number of deaths due to furious rabies as estimated by Million Deaths Study in 2012 was 12700 ^6^. IDSP (Integrated Disease Surveillance Programme) reported an increase in animal bites from 4.2 million in 2012 to 7.4 million in 2018 and dogs are responsible for more than 95% of rabies deaths ^7^. At these rates, India contributes approximately one third of global rabies deaths annually. The disease mainly affects people belonging to lower socio-economic categories, and children in the age group of 5–15 years in the country ^8^.

Despite presence of the National Rabies Control Programme (NRCP), incidence of rabies has remained stagnant and grossly under-reported ^9^. The true burden of disease is not reflected in hospital data due to issues in reporting and community-based systems are considered better for rabies surveillance in India ^10^. There is serious need of improved reporting systems to address the lack of accurate data and its verification in a number of regions in the country ^10, 11^.

This systematic review protocol attempts to measure the magnitude of the epidemiological, economic and humanistic burden of dog-mediated rabies in India. It has been planned to highlight key evidence gaps, precise measurements and utilization decisions to enable policymakers to frame the best health practice solutions. A preliminary search on PROSPERO, MEDLINE, Cochrane Database of Systematic Reviews and JBI Database of Systematic Reviews and Implementation Reports was conducted, and no current or ongoing systematic reviews on the topic were identified to the best of our knowledge.

## Protocol

### Methods/design

The methods of this systematic review have been developed and reported in compliance with the Preferred Reporting Items for Systematic Reviews and Meta-Analysis Protocols (PRISMA-P) ^12^ (see *Reporting guidelines* for a completed checklist ^13^). In accordance with guidelines, the study protocol is registered with the International Prospective Register of Systematic Reviews (PROSPERO) with ID: CRD42020213261

### Objectives

To synthesize evidence on epidemiological, humanistic and economic burden of dog bites and dog-mediated rabies and its complications in India

### Research question(s)

What is the epidemiological, humanistic and economic burden of dog bites and dog-mediated rabies and its complications in India?

### Eligibility criteria

***Population.*** This systematic review will include rabies patients and human dog-bite victims from India irrespective of their age and gender.

***Outcomes***

- *Epidemiological outcomes:* Prevalence, category and socio-demographic trends of dog bites; clinical and epidemiological profile of victims, morbidity and; incidence of rabies.
- *Humanistic outcomes:* Utility and health-related quality of life (HRQoL) measurements associated with dog bites and rabies.
- *Economic outcomes:* Various costs including direct and indirect costs and resources utilized in association with dog-bite and rabies.

### Study design

The following study designs will be included:

- For epidemiological outcomes, Randomized Control Trials (RCTs) with comparator arm, cohort and cross-sectional studies will be included.
- For humanistic outcomes, RCTs with comparator arm, case-control, cohort and cross-sectional studies reporting patient reported HRQoL and other utility and humanistic outcomes will be included.
- For economic outcomes, partial economic evaluation such as cost, cost of illness and resource utilization analyses; and full economic evaluation such as cost-effectiveness, cost utility, cost minimization, and cost-benefit analyses studies will be included.

Studies without the relevant data on the outcomes of interest, such as rabies caused due to other animals, in languages other than English, not having an Indian context, and conducted on mammals other than humans will be excluded. The modelling studies reporting the outcomes of interest in Indian context will also be screened for inclusion criteria.

### Search strategy

The search strategy will include identification of both published and unpublished studies. A preliminary limited search of MEDLINE was conducted to identify articles on the topic. The words present in the titles and abstracts of the relevant articles, and the index terms used to describe the articles were used to develop a full search strategy. The search strategy, including all identified keywords and index terms, will be adapted for each included information source. Controlled vocabularies (e.g. Medical Subject Heading terms) to identify synonyms were used. The MEDLINE search strategy is available as *Extended data* ^13^.

Additional databases to be searched include EMBASE, Cochrane Central, PROQUEST, and Shodhganga. An Advisory Board comprising of researchers and experts working in the field of rabies in India will be established for guidance on the identification of grey literature such as technical reports by the Ministry of Health and Family Welfare and other institutions, Masters/PhD thesis etc., and their opinion. Administrative data from Integrated Disease Surveillance Programme (IDSP), Health Management Information System (HMIS) and Central Bureau of Health Intelligence (CBHI) will be searched and analyzed ^14^. The reference list of all studies selected for critical appraisal will be screened extensively for additional studies. There is no specific timeline for the literature search. All the articles identified till the last date of literature search will be subjected to screening for inclusion criteria. 

### Study selection

All identified studies will be pooled and uploaded into Rayyan QCRI software and duplicates will be first removed. Titles and abstracts will then be screened and assessed against the inclusion criteria for the review by two independent reviewers (AR & DJ) using Rayyan QCRI software ^15^. The eligible studies after the initial screening will be retrieved in full and will be assessed in detail against the inclusion criteria by two independent reviewers (AR & DJ). Reasons for exclusion of full-text studies unable to meet the inclusion criteria will be recorded and reported in the final analysis. Any disagreements between the reviewers at any stage of the selection process will be resolved through discussion, or in consultation with a third reviewer (OB). The results of the search will be reported in full in the final systematic review and presented as per Preferred Reporting Items for Systematic Reviews and Meta-analyses (PRISMA) guidelines ^16^.

### Assessment of methodological quality and risk of bias

- *Epidemiological burden:* Eligible studies will be critically appraised for methodological quality using STROBE checklist for cohort and cross-sectional studies ^17, 18^ and; the JBI standardized critical appraisal instrument for RCTs ^19^.
- *Humanistic burden:* The methodological quality of the eligible studies for the included HRQoL measures will be critically appraised by using Consensus-based Standards for the selection of health Measurement Instruments (COSMIN) checklist ^20^.
- *Economic burden:* Eligible partial economic evaluation and full economic evaluation will be critically appraised by using Consensus Health Economic Criteria (CHEC) list and Consolidated Health Economics Evaluation Reporting Standards (CHEERS) checklist respectively ^21, 22^.

Epidemiological and/or mathematical modeling studies will be assessed using Optional scoring checklist for the assessment of the degree of model validation for modeling studies ^23^.

Annexure 1 provides the extraction forms for the various checklists used for the included studies ( *Extended data* ^13^). The results of the critical appraisal will be reported in narrative and tabular formats.

### Data extraction

The following data will be extracted with regards to epidemiological burden: publication date and details, authors, location, setting, study population, study period and sample size. The data extracted will also include specific details about the condition, populations, study methods and proportions of interest to the review question and specific objectives. If studies did not specify the exact years of study, the year of publication will be used. Annexure 2 provides data extraction tool for epidemiological data ( *Extended data* ^13^).

For the humanistic burden, different disease-specific HRQoL measures will be extracted. For each questionnaire, the dimensions of HRQoL that are assessed will be identified. Annexure 3 provides data extraction tool for humanistic burden data ( *Extended data* ^13^).

For the economic burden, the form will be structured based on the format and guidelines used to produce structured abstracts of economic evaluations for inclusion in the CCEMG and data items included in published studies ^24^. Annexure 4 provides data extraction tool for economic data ( *Extended data* ^13^).

The data specific to dog-bites and dog-mediated rabies will be extracted from the studies reporting data from all animal bites. In cases of uncertainty or missing data, the corresponding authors will be contacted for additional information, missing or additional data. Any disagreements that arise between the reviewers will be resolved through discussion, or with a third reviewer (OB). 

### Data synthesis

- *Epidemiological burden:* Data extracted from the included studies will, where possible (e.g. studies using uniform case definitions, the same measures of outcome, context and approaches), will be pooled and proportional meta-analysis will be conducted using Metafor package in R.
- *Humanistic burden:* Depending on the quantity, quality and nature of the papers identified, humanistic outcome results will be subjected to a narrative and tabulated summary.
- *Economic burden:* Depending on quantity, quality and nature of the economic papers identified, economic results will be subjected to a narrative summary, or sorting in tables by comparisons/outcomes.

### Statistical analysis

If meta-analysis is possible, data from the included studies for epidemiological burden will be transformed using a Logit transformation or double arcsine transformation to calculate the weighted summary of proportion (pooled incidence and prevalence) under a random effect model. The effect size will be expressed as a proportion with 95% confidence intervals around the summary estimate. Heterogeneity will be assessed using the Chi-squared, Tau-squared and I-squared tests. To explore potential sources of heterogeneity from the included studies, characteristics likely to modify incidence/ prevalence estimates will be considered for subgroup analysis. The following probable subgroups will be analyzed (where possible): gender, age groups, socio-economic status and region. Sensitivity analyses will be performed to explore the impact of individual studies on the overall calculated estimates. This will be performed by investigating whether dropping or adding primary studies with slightly non-standard disease definitions will make a difference. Where statistical pooling in a meta-analysis is not possible due to heterogeneity, the findings will be presented in a narrative form including tables and figures to aid in data presentation. Sources of heterogeneity and reason for which it is determined to be inappropriate to pool data will be specified in the systematic review report.

Data related to HRQoL and utility will be presented in narrative and tabulated summary according to the tools and measures used in the available studies as per various probable sub groups.

Data on costs and resources utilized will be presented in narrative summary and tabulated form to shed light on whether there are differences as per various possible subgroups including category of exposure, prophylaxis, distance from health facility, type of health facility and socio-demographic differences. The available unit cost data will be tabulated along with reporting of price year. The costs will be converted in 2020 International Dollars’ value using implicit price deflators for Purchasing Power Parities as recommended by CCEMG for greater transparency and comparability across studies ^24, 25^.

The publication bias will be assessed through generation of a funnel plot if at least 10 studies are included in meta-analysis. The symmetry of Funnel plot will be tested by Egger test ^26, 27^.

## Discussion

This systematic review will be performed to critically examine relevant literature on epidemiological, humanistic and economic burden of dog-mediated rabies in the Indian context. The aim is to identify and report the epidemiological burden of dog bites, dog bite victims and rabies; direct and indirect costs associated with prophylaxis of dog bites and; utility and other humanistic outcomes in rabies in Indian context. Understanding these parameters could help policy makers to understand the burden of the illness in the country and will aid in proper allocation of scarce resources and funding. This will also help in proper formulation and effective implementation of the national program. This review will also help in implementation research in India. Limitation of the review, its implications and suggestions for future research will also be provided.

## Data availability

### Underlying data

No data is associated with this article

### Extended data

Figshare: Extended Data Set: The epidemiological, humanistic and economic burden of dog-mediated rabies in India_a systematic review protocol.pdf, https://doi.org/10.6084/m9.figshare.13385474.v1 ^13^.

This project contains following extended data:

- MEDLINE Search Strategy
- Critical appraisal checklist
- Data extraction tool for epidemiological burden studies
- Data extraction tool for humanistic burden studies
- Data extraction tool for economic burden studies

### Reporting guidelines

Figshare: PRISMA-P checklist for ‘The epidemiological, humanistic and economic burden of dog-mediated rabies in India: a systematic review protocol’, https://doi.org/10.6084/m9.figshare.13385474.v1 ^13^.

Data are available under the terms of the Creative Commons Attribution 4.0 International license (CC-BY 4.0).
