# Supplementary material for: Epidemiological, humanistic and economic burden of dog-mediated rabies in India: a systematic review protocol
Source: F1000Res. 2021 May 12;10:32. Originally published 2021 Jan 18. [Version 2] doi: 10.12688/f1000research.28454.2 (PMC8063559; doi:10.12688/f1000research.28454.2)
Supplement: Peer review history

# Peer review: Reviewer response for version 2

**DOI:** 10.5256/f1000research.56133.r85248

**Published:** 2021-05-12

**Authors:** Sumon Ghosh

**Affiliations:** 1 Infectious Diseases Division, International Centre for Diarrhoeal Disease Research, Bangladesh (icddr,b), Dhaka, Bangladesh

I am satisfied that the authors have addressed the points I made in my first review. I do not have any further comments.

Is the study design appropriate for the research question?

Yes

Is the rationale for, and objectives of, the study clearly described?

Yes

Are sufficient details of the methods provided to allow replication by others?

Yes

Are the datasets clearly presented in a useable and accessible format?

Not applicable

Reviewer Expertise:

My research focuses on surveillance of avian influenza, rabies control, and antimicrobial resistance in Bangladesh.

I confirm that I have read this submission and believe that I have an appropriate level of expertise to confirm that it is of an acceptable scientific standard.

# Peer review: Reviewer response for version 1

**DOI:** 10.5256/f1000research.31493.r83097

**Published:** 2021-04-13

**Authors:** Sumon Ghosh

**Affiliations:** 1 Infectious Diseases Division, International Centre for Diarrhoeal Disease Research, Bangladesh (icddr,b), Dhaka, Bangladesh

This Study Protocol is well-written which includes all the information necessary to conduct a systematic review and I think very useful contribution to the literature. The article is detailed and written using a standard format to write systematic review protocol. This protocol clearly spelled out the magnitude of the epidemiological, economic and humanistic burden of dog-mediated rabies in a part of the world where rabies has remained stagnant and grossly under-reported.

 I have only a few minor comments:

1. The author did not mention the timeline for selection of the articles.
2. Mention about the inclusion of modelling studies reporting the outcomes of interest in Indian context conducted at international/regional level.
3. Will the data related to dog bites be reported from the studies reporting bites from all animals?

Is the study design appropriate for the research question?

Yes

Is the rationale for, and objectives of, the study clearly described?

Yes

Are sufficient details of the methods provided to allow replication by others?

Yes

Are the datasets clearly presented in a useable and accessible format?

Not applicable

Reviewer Expertise:

My research focuses on surveillance of avian influenza, rabies control, and antimicrobial resistance in Bangladesh.

I confirm that I have read this submission and believe that I have an appropriate level of expertise to confirm that it is of an acceptable scientific standard.

# Response

**Published:** 2021-04-13

**Authors:** Abhishek Royal

**Affiliations:** Universitas Gadjah Mada, Indonesia

Thanks a lot for providing your valuable comments to this article. We have incorporated following changes to the article as per your suggestions:

1. The modelling studies reporting the outcomes of interest will also be searched and will be included after screening for inclusion criteria.
2. There is no specific timeline for the literature search. All the articles identified till the last date of literature search will be subjected to screening for inclusion criteria in this systematic review.
3. The data specific to dog-bites and dog-mediated rabies will be extracted from the studies reporting data from all animal bites.

# Peer review: Reviewer response for version 1

**DOI:** 10.5256/f1000research.31493.r80932

**Published:** 2021-03-17

**Authors:** Koen K.A. Van Rompay

**Affiliations:** 1 California National Primate Research Center, University of California, Davis, Davis, CA, USA

The article is well-written and I do not have any major comments.

 I have several minor comments/suggestions/corrections:

- In the Abstract, Background section: The statement, *"The disease is almost fatal after the onset of symptoms"* should be corrected to indicate that it it almost always fatal.
- Introduction: The word "of" is missing in the sentence, *"...more than 95% of human cases...".*
- Introduction, last sentence: I suggest specifying that here with pre-exposure prophylaxis one means vaccination.
- Protocol - Eligibility criteria - Population: one can add "India".
- Under Eligibility criteria, in the section "Outcomes", fix the spelling error in "health-related". 

Is the study design appropriate for the research question?

Yes

Is the rationale for, and objectives of, the study clearly described?

Yes

Are sufficient details of the methods provided to allow replication by others?

Yes

Are the datasets clearly presented in a useable and accessible format?

Not applicable

Reviewer Expertise:

I work in animal models of infectious diseases (so have limited/moderate expertise in epidemiology).

I confirm that I have read this submission and believe that I have an appropriate level of expertise to confirm that it is of an acceptable scientific standard.

# Response

**Published:** 2021-04-13

**Authors:** Abhishek Royal

**Affiliations:** Universitas Gadjah Mada, Indonesia

Thanks a lot for providing your valuable comments for this articles. We have incorporated following changes as per your suggestions:

1. Rabies has been mentioned as an ‘almost always fatal’ disease after the onset of symptoms.
2. The pre-exposure prophylaxis has been mentioned in the form of vaccination for high risk individuals.
3. The participants has been mentioned to be based in India. 
4. The grammatical and spelling errors has been corrected.
